# Morphology and genetic characterization of *Physaloptera sibirica* Petrow & Gorbunov, 1931 (Spirurida: Physalopteridae), from the hog-badger *Arctonyx collaris* Cuvier (Carnivora: Mustelidae), with molecular phylogeny of Physalopteridae

**DOI:** 10.1186/s13071-023-05838-6

**Published:** 2023-07-07

**Authors:** Hui-Xia Chen, Jia-Lu Zeng, Yun-Yun Gao, Dong Zhang, Yang Li, Liang Li

**Affiliations:** 1 Hebei Collaborative Innovation Center for Eco‐Environment; Hebei Key Laboratory of Animal Physiology, Biochemistry and Molecular Biology, College of Life Sciences, Hebei Normal University, 050024 Shijiazhuang, Hebei Province People’s Republic of China; 2 Hebei Research Center of the Basic Discipline Cell Biology; Ministry of Education Key Laboratory of Molecular and Cellular Biology, 050024 Shijiazhuang, Hebei Province People’s Republic of China; 3 Shenzhen Branch, Guangdong Laboratory of Lingnan Modern Agriculture, Genome Analysis Laboratory of the Ministry of Agriculture and Rural Affairs, Agricultural Genomics Institute at Shenzhen, Chinese Academy of Agricultural Sciences, Shenzhen, Guangdong 518120 People’s Republic of China; 4 School of Ecology and Nature Conservation, Beijing Forestry University, Beijing, 100083 People’s Republic of China

**Keywords:** Nematoda, Physalopteridae, Wildlife, *Arctonyx collaris*, Integrative taxonomy, Genetic data, Molecular phylogeny

## Abstract

**Background:**

Nematodes of the family Physalopteridae (Spirurida: Physalopteroidea) commonly parasitize the alimentary canal of all major vertebrate groups. However, many physalopterid species are not adequately described, especially regarding the detailed morphology of the cephalic end. The current genetic database for *Physaloptera* species is still very limited, which seriously hampers molecular-based species identification. Additionally, the systematic status of some genera and the evolutionary relationships of the subfamilies in the Physalopteridae remain under debate.

**Methods:**

New morphological data for *Physaloptera sibirica* was gathered using light and scanning electron microscopy based on newly collected specimens from the hog badger *Arctonyx collaris* Cuvier (Carnivora: Mustelidae) in China. Six different genetic markers, including nuclear small ribosomal DNA (18S), large ribosomal DNA (28S) and internal transcribed spacer (ITS), mitochondrial cytochrome c oxidase subunit 1 (*cox*1) and subunit 2 (*cox*2), and the 12S small subunit ribosomal RNA gene of *P. sibirica* were sequenced and analyzed for the first time to our knowledge. Additionally, to construct a basic molecular phylogenetic framework for the Physalopteridae, phylogenetic analyses were performed based on the *cox*1 and 18S + *cox*1 genes using maximum likelihood (ML) and Bayesian inference (BI) methods.

**Results:**

Scanning electron microscopy (SEM) observation displayed the details of the cephalic structures, deirids, excretory pore, caudal papillae, vulva, phasmids and egg of *P. sibirica* for the first time to our knowledge. Pairwise comparison of the sequences obtained for *P. sibirica* did not reveal intraspecific divergence regarding the 18S, 28S, *cox*1 and 12S genetic markers and a low level of divergence in the ITS (0.16%) and *cox*2 (2.39%) regions. Maximum likelihood and Bayesian inference analyses showed that the representatives of Physalopteridae formed two major clades (species of Physalopterinae + Thubunaeinae parasitic in terrestrial vertebrates and Proleptinae only occurring in marine or freshwater fishes). *Turgida turgida* was found nested among representatives of *Physaloptera*. *Physaloptera sibirica* clustered together with *P. rara. Physalopteroides* sp. (Thubunaeinae) formed a sister relationship to the physalopterine *Abbreviata caucasica*.

**Conclusions:**

*Physaloptera sibirica* was redescribed, which is the fourth nematode parasite reported from the hog badger *A. collaris*, and *A. collaris* represents a new host for *P. sibirica*. The phylogenetic results challenged the validity of the subfamily Thubunaeinae and of the genus *Turgida* and supported dividing the family Physalopteridae into two subfamilies, Physalopterinae and Proleptinae. However, we do not make any immediate systematic changes in the Physalopteridae, because a more rigorous study with broader representation of the Physalopteridae is required. These present findings contribute to morphologically identifying *P. sibirica* more accurately and provide new insights into the systematics of the Physalopteridae.

**Graphical Abstract:**

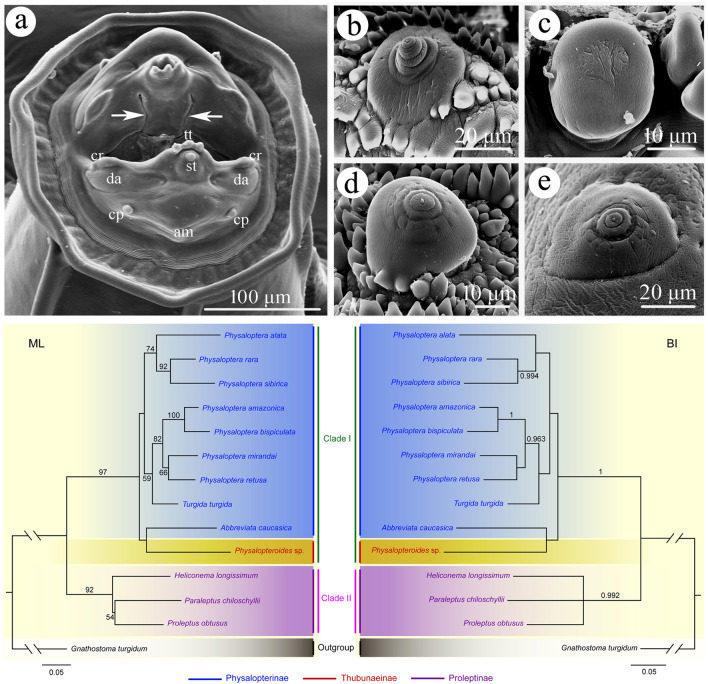

## Background

Nematodes of the family Physalopteridae (Spirurida: Physalopteroidea) commonly parasitize the alimentary canal of all major vertebrate groups [1–5]. According to the current classification, Physalopteridae is divided into three subfamilies, Physalopterinae, Proleptinae and Thubunaeinae [6]. Chabaud & Bain (1994) [7] speculated that the Proleptinae are morphologically closer to the Physalopterinae based on the cephalic structures; however, the evolutionary relationships of the representatives of these subfamilies remain unclear.

The genus *Physaloptera*, with over 100 nominal species reported from various amphibians, reptiles, birds and mammals worldwide, is the largest group in the Physalopteridae [8–10]. However, many species of *Physaloptera* are not adequately described, especially regarding the detailed morphology of the cephalic structure. Moreover, the current genetic database for *Physaloptera* species is still very limited. In *Physaloptera*, only 11 species have been genetically sequenced (many of these genetic data are still unpublished), which seriously hampers the molecular-based species identification and phylogeny of this group.

The greater hog badger *Arctonyx collaris* Cuvier (Carnivora: Mustelidae) is a terrestrial mammal, mainly distributed in Central and Southeast Asia, including Thailand, India, Bangladesh and P.R. China. This species is listed as vulnerable in the IUCN Red List of Threatened Species (https://www.iucnredlist.org/species/70205537/45209459), because the global population is thought to be declining because of over-hunting and the destruction of its habitat [11]. There is currently very little information on the nematode fauna of *A. collaris*. To date, only three nematode species have been reported from it, including *Uncinaria stenocephala* (Railliet, 1884) (Rhabditida: Ancylostomatidae), *Toxocara vajrasthirae* Sprent, 1972 (Ascaridida: Ascarididae) and *Tetragomphius arctonycis* Jansen, 1968 (Rhabditida: Ancylostomatidae) [12–14].

In the present study, some physalopterid nematodes were collected from *A. collaris* in China. The detailed morphology of these nematode specimens was studied using light and scanning electron microscopy, and the molecular characterization of the nuclear small subunit ribosomal DNA (18S), internal transcribed spacer (ITS) and large subunit ribosomal DNA (28S), and mitochondrial cytochrome c oxidase subunit 1 (*cox*1) and subunit 2 (*cox*2) and 12S regions were sequeced and analyzed to accurately identify them to species level. Additionally, to clarify the evolutionary relationships of the representatives of the Physalopterinae, Proleptinae and Thubunaeinae in the Physalopteridae and construct a basic molecular phylogenetic framework for this group, phylogenetic analyses based on the 18S + *cox*1 and *cox*1 sequence data using maximum likelihood (ML) and Bayesian inference (BI) were performed.

## Methods

### Parasite collection

A road-killed hog badger *A. collaris* Cuvier (Carnivora: Mustelidae) in Huangniupu (106.786E, 34.246N), Baoji city, Shaanxi Province, was opportunistically examined for parasites. The hog badger was a male with 50–60 cm body length. Only some nematode parasites (26 specimens) were isolated from the stomach of this host and were stored in 80% ethanol for further study.

### Morphological observation

For light microscopical studies, nematodes were cleared in glycerine. Drawings were made with a Nikon microscope drawing attachment. For scanning electron microscopy (SEM), the cephalic and posterior ends of nematodes were fixed in 4% formaldehyde solution, post-fixed in 1% OsO4, dehydrated via an ethanol series and acetone, and then critical point dried. Samples were coated with gold and examined using a Hitachi S-4800 scanning electron microscope at an accelerating voltage of 20 kV. Measurements (range followed by mean in parentheses) are given in millimeters (mm) unless otherwise stated. Voucher specimens were deposited at the College of Life Sciences, Hebei Normal University, Hebei Province, China.

### Molecular procedures

Three nematode specimens (1 male and 2 females) were randomly chosen for molecular analyses. Genomic DNA from each sample was extracted using a Column Genomic DNA Isolation Kit (Shanghai Sangon, China) according to the manufacturer’s instructions. The primers used for amplifying different target regions by polymerase chain reaction (PCR) in the present study are provided in Table 1. The cycling conditions were as described previously [15]. PCR products were checked on GoldView-stained 1.5% agarose gels and purified with Column PCR Product Purification Kit (Shanghai Sangon, China). Sequencing for each sample was carried out for both strands. The DNA sequences obtained herein using the PCR primers were aligned using ClustalW2 and compared (using the algorithm BLASTn) with those available in the National Center for Biotechnology Information (NCBI) database (http://www.ncbi.nlm.nih.gov). The 18S, 28S, ITS, *cox*1, *cox*2 and 12S sequence data obtained herein were deposited in the GenBank database (http://www.ncbi.nlm.nih.gov).

**Table 1:** Detailed information on primers used for amplifying different target regions by polymerase chain reaction (PCR) in the present study

Gene	Primer name	Primer sequence (5′ → 3′)	References
18S	18S-F	CGCGAATRGCTCATTACAACAGC	[46]
	18S-R	GGGCGGTATCTGATCGCC	
28S	28S-F	AGCGGAGGAAAAGAAACTAA	[47]
	28S-R	ATCCGTGTTTCAAGACGGG	
ITS1-5.8S	SS1	GTTTCCGTAGGTGAACCTGCG	[48]
	SS2R	AGTGCTCAATGTGTCTGCAA	
ITS2	NC13	ATCGATGAAGAACGCAGC	[48]
	NC2	TTAGTTTCTTTTCCTCCGCT	
*cox*1	NTF	TGATTGGTGGTTTTGGTAA	[49]
	NTR	ATAAGTACGAGTATCAATATC	
*cox*2	COII-F	AATTTTAATTGTAGTCTTTTGTTTGG	[50]
	COII-R	CTATGATTAGCACCACAAATC	
12S	12S-F	GTTCCAGAATAATCGGCTA	[51]
	12S-R	ATTGACGGATGGTTTGTACC	

### Phylogenetic analyses

Phylogenetic reconstructions were performed based on the 18S + *cox*1 and *cox*1 sequence data using the maximum likelihood (ML) and Bayesian inference (BI) criteria implemented within IQ-TREE and MrBayes [16, 17], respectively. *Gnathostoma turgidum* Stossich, 1902 (Spirurida: Gnathostomatidae) was treated as the outgroup according to the previous studies [18, 19]. The ingroup included 16 physalopterid species representing seven genera belonging to three subfamilies, Physalopterinae, Proleptinae and Thubunaeinae. Detailed information on nematode species included in the phylogenetic analyses is provided in Table 2. We used a built-in function in IQTREE to select a best-fitting substitution model for the sequences according to the Bayesian information criterion [20]. The TIM3 + F + I + G4 model was identified as the optimal nucleotide substitution model for both 18S + *cox*1 and *cox*1 sequence data. Reliabilities for maximum likelihood inference were tested using 1000 bootstrap replications, and Bayesian information criterion analysis was run for 1 × 10^7^ MCMC generations, sampling a tree at every 1000 generations. The first 25% trees were treated as “burn-in.” Reliabilities for ML tree were tested using 1000 bootstrap replications, and BI tree was tested using 10 million generations. In the ML tree, bootstrap support (BS) values ≥ 80 were considered to constitute strong branch support, whereas BS values ≥ 50 and < 80 were considered to constitute moderate branch support. In the BI tree, Bayesian posterior probabilities (BPP) ≥ 0.98 were considered to constitute strong branch support, whereas BPP values ≥ 0.95 and < 0.98 were considered to constitute moderate branch support.

**Table 2:** Species of Physalopteridae with their detailed information on genetic data included in the phylogenetic analyses

Species	Host	Locality	GenBank ID for 18S region	GenBank ID for *cox*1 region	References
Ingroup					
Physalopterinae					
* Abbreviata caucasica*	*Pan troglodytes verus* (Mammalia: Primates)	Senegal	MN956824	MT231294	[52]
* Physaloptera alata*	*Accipiter gentilis* (Aves: Falconiformes); *Hieraaetus pennatus* (Aves: Accipitriformes)	Germany; Portugal	AY702703	MZ391893	Unpublished
* P. amazonica*	*Proechimys gardneri* (Mammalia: Rodentia)	Brazil	MK312472	MK309356	[18]
* P. bispiculata*	*Nectomys squamipes* (Mammalia: Rodentia)	Brazil	KT894817	KT894806	Unpublished
* P. hispida*	*Sigmodon hispidus* (Mammalia: Rodentia)	USA	–	MH782845	[53]
* P. mirandai*	*Metachirus nudicaudatus* (Mammalia: Didelphimorphia)	Brazil	KT894816	KT894805	Unpublished
* P. rara*	*Canis lupus familiaris* (Mammalia: Carnivora)	USA	MH938367	MH931178	Unpublished
* P. retusa*	*Tupinambis teguixin* (Reptilia: Squamata)	Brazil	KT894814	KT894803	Unpublished
* P. sibirica*	*Arctonyx collaris* (Mammalia: Carnivora)	China	OQ846900	OQ852731	Present study
* Turgida turgida*	*Didelphis virginiana*,* D. aurita* (Mammalia: Didelphimorphia)	USA; Brazil	DQ503459	KT894808	[54]; Unpublished
* Turgida* sp.	*Didelphis virginiana* (Mammalia: Didelphimorphia)	Mexico	–	KC130680	[55]
Proleptinae					
* Heliconema longissimum*	*Anguilla japonica* (Actinopterygii: Anguilliformes)	Japan	JF803926	NC_016127	[56, 57]
* Paraleptus chiloschyllii*	*Chiloscyllium punctatum* (Elasmobranchii: Orectolobiformes)	China	OK482082	MZ958986	[58]
* Proleptus obtusus*	*Scyliorhinus canicula* (Elasmobranchii: Carcharhiniformes)	Portugal	KY411575	KY411574	[59]
Thubunaeinae					
* Physalopteroides* sp.	*Hemidactylus brooki* (Reptilia: Squamata)	India	KP338605	–	[60]
* Physalopteroides* sp.	*Sceloporus* sp. (Reptilia: Squamata)	Mexico	–	KC130709	[55]
Outgroup					
* Gnathostoma turgidum*	*Didelphis aurita* (Mammalia: Didelphimorphia)	Brazil	Z96948	KT894798	Unpublished; [61]

## Results

**Order Spirurida Railliet, 1914**

**Superfamily Physalopteroidea Railliet, 1893**

**Family Physalopteridae Railliet, 1893**

**Genus** ***Physaloptera*** **Rudolphi, 1819**

**Physaloptera sibirica Petrow & Gorbunow, 1931**

***Host***: The hog badger *Arctonyx collaris* Cuvier (Carnivora: Mustelidae).

***Locality***: Shaanxi Province, China.

***Site of infection***: Stomach.

***Specimen deposition***: 5 males, 10 females (HBNU-N-2022M1120-CL); deposited at the College of Life Sciences, Hebei Normal University, Hebei Province, China.

***Representative DNA sequences***: Representative nuclear ribosomal and mitochondrial DNA sequences were deposited in the GenBank database under accession nos. OQ846900–OQ846902 (18S), OQ846911–OQ846913 (ITS), OQ846907–OQ846909 (28S), OQ852731–OQ852733 (*cox*1), OQ867994–OQ867996 (*cox*2) and OQ846904–OQ846906 (12S).

### Description (Figs. 1, 2, 3)

**Fig. 1:**
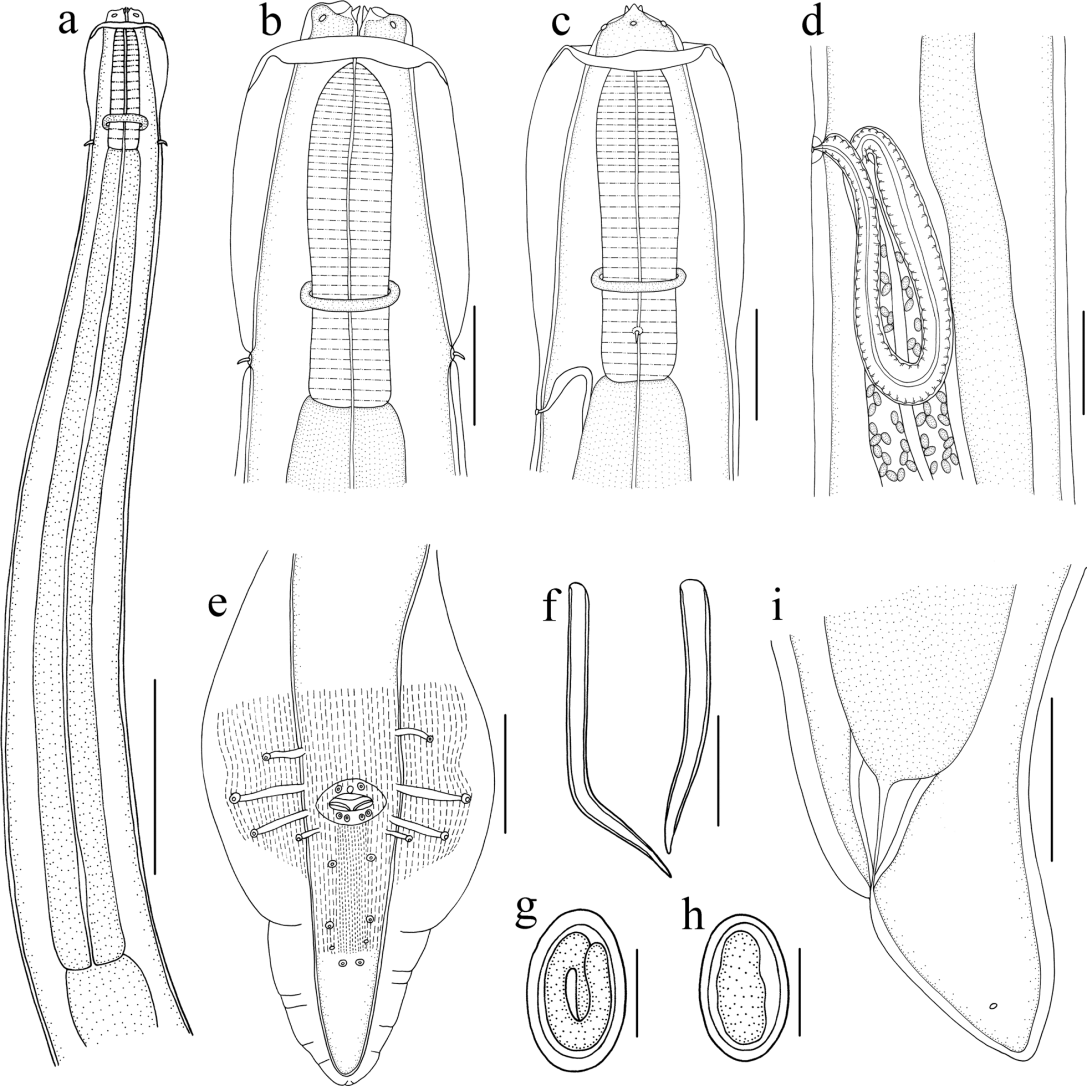
*Physaloptera sibirica* collected from *Arctonyx collaris* Cuvier (Carnivora: Mustelidae) in China. **a** Anterior part of male, ventral view. **b** Anterior end of male, ventral view. **c** Anterior end of male, lateral view. **d** Region of vulva, lateral view. **e** Posterior end of male, ventral view. **f** Spicules. **g** Egg, embryonated. **h** Egg, unembryonated. **i** Posterior end of female, lateral view. Scale bars: **a** 1000 μm; **b**, **c**, **f**: 200 μm; **d**, **e**, **i**: 500 μm; **g**, **h**: 30 μm

**Fig. 2:**
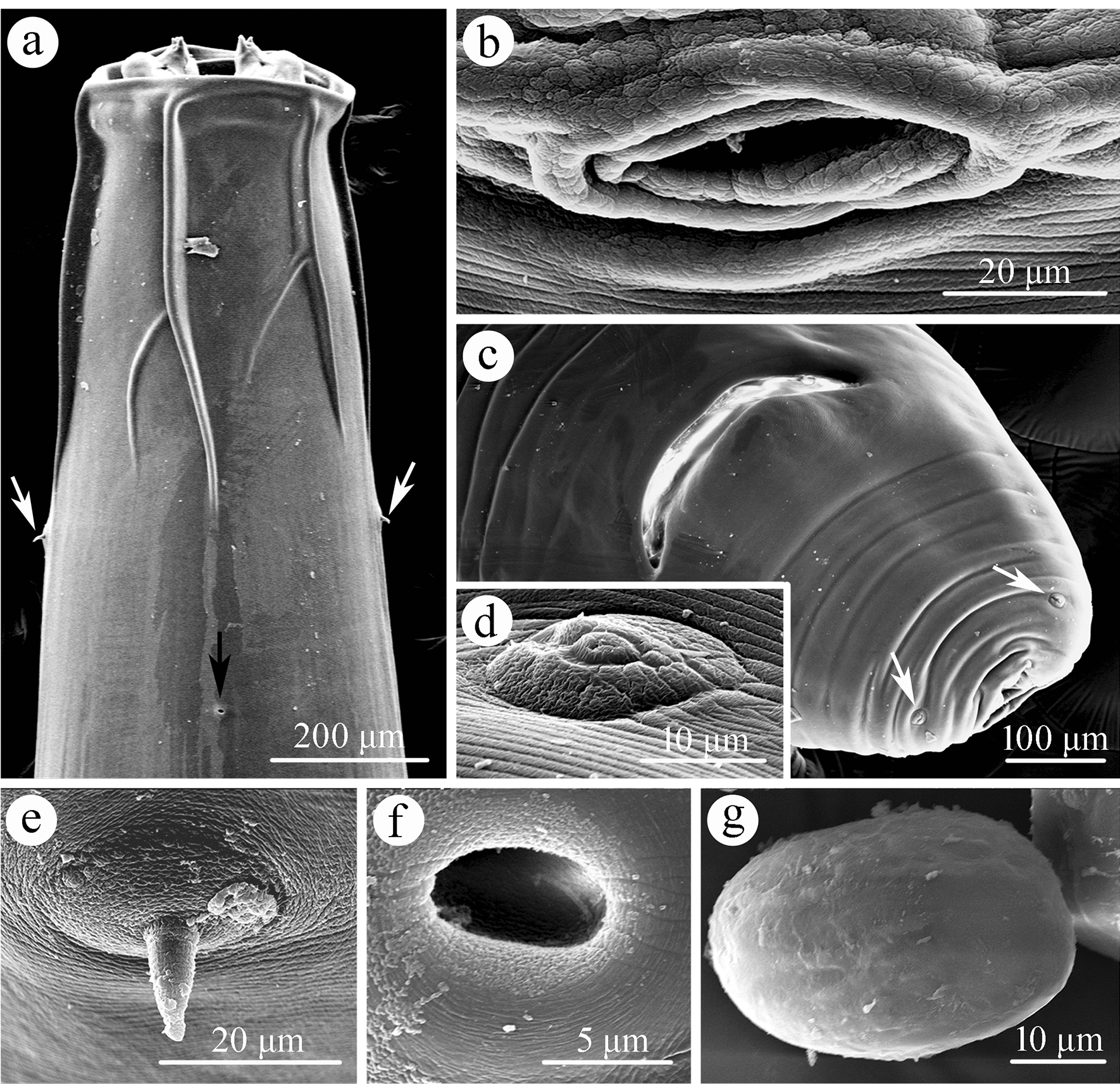
Scanning electron micrographs of *Physaloptera sibirica* collected from *Arctonyx collaris* Cuvier (Carnivora: Mustelidae) in China, female. **a** Anterior part of body (deirids indicated by white arrows, excretory pore indicated by black arrow), ventral view. **b** Magnified image of vulva. **c** Tail (phasmids arrowed), ventral view. **d** Magnified image of phasmid. **e** Magnified image of deirid. **f** Magnified image of excretory pore. **g** Magnified image of egg

**Fig. 3:**
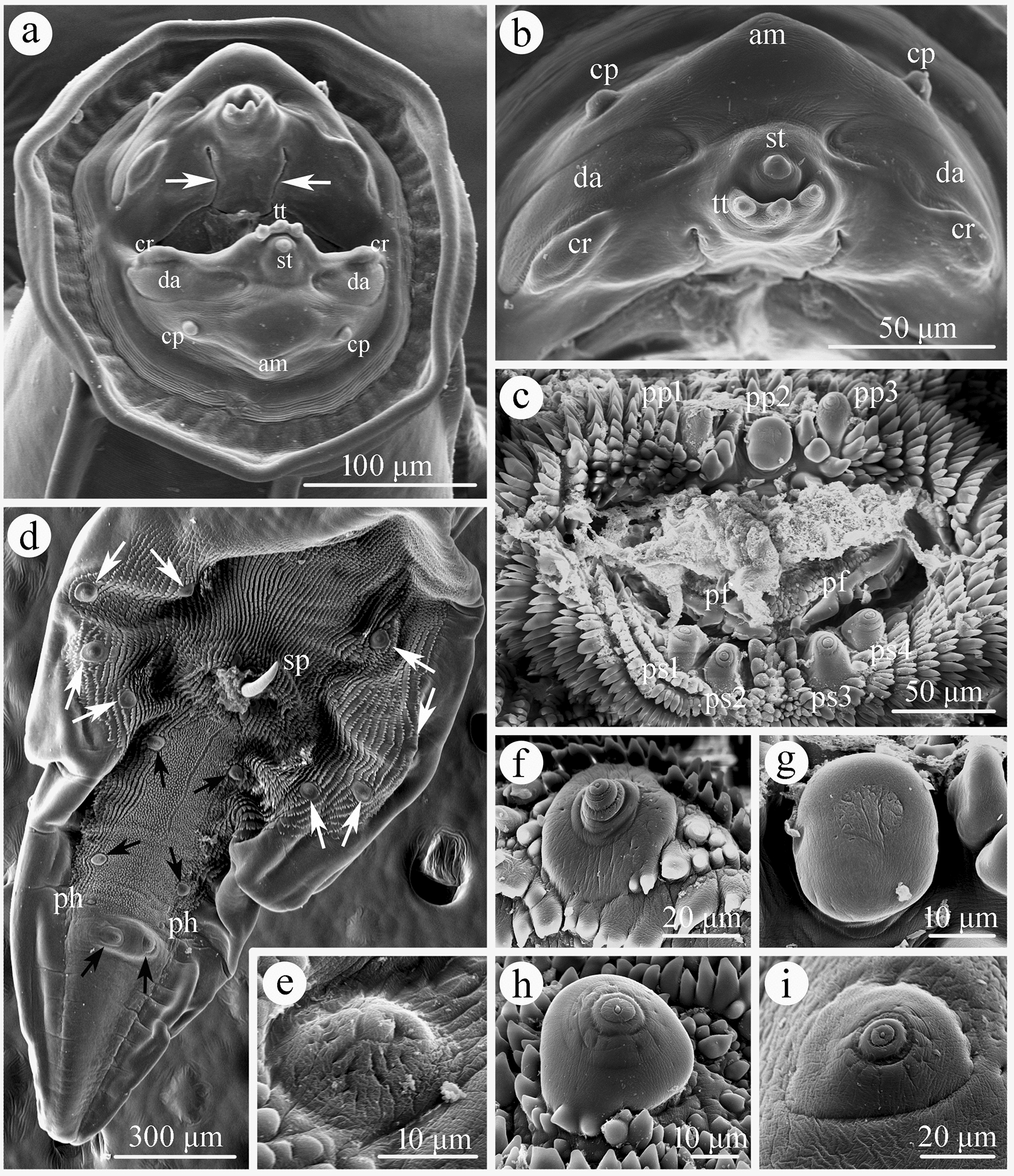
Scanning electron micrographs of *Physaloptera sibirica* collected from *Arctonyx collaris* Cuvier (Carnivora: Mustelidae) in China, male. **a** Cephalic extremity (longitudinal grooves present at internal base of pseudolabium indicated by arrows), subapical view. **b** Magnified image of lateral pseudolabium, apical view. **c** Magnified image of cloacal region, ventral view. **d** Posterior end of body (4 pairs of precloacal and postcloacal pedunculate papillae indicated by white arrows, 3 pairs of postcloacal ventral sessile papillae indicated by black arrows), ventral view. **e** Magnified image of phasmid. **f** Magnified image of second pair of precloacal pedunculate papilla. **g** Magnified image of median sessile papilla at anterior margin of cloaca. **h** Magnified image of first pair of postcloacal ventral sessile papilla. **i** Magnified image of third pair of postcloacal ventral sessile papilla. cp, submedian cephalic papillae; am, amphid; st, simple tooth; tt, tripartite tooth; da, depressed areas on each pseudolabium; cr, cuticular ridges; pp1–3, first to third pairs of sessile papillae at anterior margin of cloaca; ps1–4, first to fourth pairs of sessile papillae at posterior margin of cloaca; pf, papillose formations on posterior cloacal lip; ph, phasmids; sp, spicule

#### General

Medium-sized whitish nematodes. Cuticle thick with fine transverse striations. Cephalic collarette (vesicle) present, extending posteriorly to the level of deirids (Figs. 1a‒c, 2a). Cephalic end dome-shaped, oral aperture dorsoventrally elongate, surrounded by two lateral pseudolabia (Figs. 1a‒c, 2a, 3a, b). Each pseudolabium bearing two large submedian (dorsolateral and ventrolateral) cephalic papillae and one small amphid situated at nearly the middle of pseudolabium (Fig. 3a, b). Inner margin of each pseudolabium with one simple median conical tooth, one median tripartite tooth and two lateral cuticular ridges (Fig. 3a, b). One pair of short longitudinal grooves present at internal base of pseudolabium. Two oval depressed areas present on each pseudolabium (Fig. 3a, b). Oesophagus divided into anterior short muscular portion and long glandular portion (Fig. 1a). Nerve-ring encircles posterior muscular oesophagus (Fig. 1a‒c). Deirids well developed, spine-shaped, slightly posterior to nerve ring (Figs. 1a‒c, 2a, e). Excretory pore situated slightly posterior to junction of muscular and glandular oesophagus (Figs. 1c, 2a, f). Posterior end of body sexually dimorphic.

#### Male (based on 5 specimens)

Body 19.6–32.7 (27.8) long; maximum width 0.73–1.12 (0.94). Pseudolabia 0.08–0.12 (0.10) long, 0.14–0.19 (0.17) wide. Cephalic collarette (vesicle) 0.46–0.81 (0.65) long, 0.20–0.25 (0.23) wide. Entire oesophagus 3.46–5.37 (4.62) long, representing 14.4–18.9 (16.8) % of body length; muscular oesophagus 0.49–0.71 (0.55) long, 0.15–0.32 (0.22) in maximum width; glandular oesophagus 3.00–4.88 (4.07) long, 0.29–0.39 (0.34) in maximum width; length ratio of two parts of oesophagus 1: 6.15–9.52 (1: 7.45). Nerve ring, deirids and excretory pore 0.44–0.56 (0.50), 0.51–0.73 (0.63) and 0.76–0.93 (0.83) from cephalic extremity, respectively. Spicules similar in shape, with subpointed distal end, unequal in length; left spicule relatively long, 0.54–1.27 (1.00) in length, representing 2.38–3.97 (3.28) % of body length; right spicule short, 0.44–1.22 (0.87) long, representing 1.94–3.82 (2.83) % of body length; spicule (right: left) ratio 1/1.04–1.25 (1/1.18) (Fig. 1f). Gubernaculum absent. Posterior end of body spirally coiled ventrally. Posterior expansion of cuticle forming spade-like caudal bursa, ornamented ventrally with numerous longitudinal cuticular tubercles (Fig. 3d) and supported by four pairs of subventral pedunculate papillae (2 pairs precloacal, 2 pairs postcloacal) (Figs. 1e, 3f). Anterior margin of cloaca with three sessile papillae (median papilla tabular, usually larger than lateral papillae), posterior margin of cloaca with two pairs of sessile papillae (Figs. 1e, 3c, g). Three additional pairs of postcloacal ventral sessile papillae present (Figs. 1e, 3d, h, i). Tail 0.88–1.93 (1.44) long, with rounded tip (Figs. 1e, 3d). Phasmids situated between second and third pairs of postcloacal ventral papillae (Figs. 1e, 3d, e).

#### Female (based on 10 specimens)

Body 25.0–51.5 (37.7) long; maximum width 1.00–1.59 (1.26). Pseudolabia 0.09–0.14 (0.12) long, 0.15–0.21 (0.18) wide. Cephalic collarette (vesicle) 0.44–0.83 (0.60) long, 0.26–0.39 (0.32) wide. Entire oesophagus 4.39–7.32 (5.67) long, representing 12.2–17.6 (15.4) % of body length; muscular oesophagus 0.49–0.73 (0.56) long, 0.15–0.27 (0.20) in maximum width; glandular oesophagus 3.90–6.59 (5.11) long, 0.34–0.49 (0.42) in maximum width; length ratio of two parts of oesophagus 1: 8.00–10.5 (1: 9.05). Nerve ring, deirids and excretory pore 0.46–0.63 (0.53), 0.51–0.85 (0.68) and 0.63–1.10 (0.85) from anterior extremity, respectively. Vulva slightly protruding, situated 4.10–9.81 (6.46) from cephalic extremity, at 13.9–21.4 (17.4) % of body length (Figs. 1d, 2b). Vagina long, muscular, initially directed posteriorly from vulva and then oriented anteriorly; uteri didelphic with two uterine branches (Fig. 1d). Eggs oval, unembryonated or embryonated, thick-shelled, with smooth surface, 0.04–0.05 (0.05) × 0.02–0.04 (0.03) (*n* = 20) (Figs. 1g, h, 2g). Posterior end of body almost straight, not spirally coiled ventrally. Tail 0.37–0.73 (0.49) long, with roughly rounded tip (Figs. 1i, 2c). A pair of small lateral phasmids present at base of tail tip (Figs. 1i, 2c, d).

### Remarks

Petrow & Gorbunow (1931) [21] described *P. sibirica* from the Eurasian badgers *Meles meles amurensis* (originally as *Nyctereutes amurensis*) and red fox *Vulpes vulpes* from the Far East, Siberia, Uzbekistan and Tatarstan. Subsequently, *P. sibirica* was reported from various mammals in the Palaearctic region [22–30]. However, only a few previous studies [2, 24, 25] provided the morphological characters of this species after the original description.

The morphology and measurements of our material are almost identical to the original description of *P. sibirica* and some other studies [2, 21, 24, 25] regarding several features, including the morphology of the pseudolabia, position of the nerve ring and excretory pore, length of the oesophagus (including muscular and glandular portions), morphology and length of the spicules and tail, number and arrangement of caudal papillae, position of the vulva and size of the eggs (see Table 3 for details). Therefore, we consider our newly collected material conspecific with *P. sibirica*. However, the body length of males in the original description [21] and Li & Zhu’s (1980) [25] material is distinctly smaller than in our specimens and Quentin & Biocca’s (1976) [24] description. Moreover, the ratio of the left and right spicules in Quentin & Biocca’s (1976) [24] material is larger than that of our specimens. The above-mentioned morphometric differences should be considered as intraspecific variability, possibly owing to the different hosts, geographical locations or infection levels. Petrow & Gorbunow (1931) [21] stated that there were 4–5 pairs of pedunculate papillae in the original description, and Skrjabin & Sobolev (1964) [2] reported only five pairs of pedunculate papillae in their redesciption, but they both considered the presence of six pairs of postcloacal sessile papillae in their material. However, the subsequent [24, 25] and present study all observed only four pairs of pedunculate papillae (2 pairs precloacal, 2 pairs postcloacal). Additionally, these two previous studies [2, 21] both erroneously treated the phasmids as one pair of postcloacal sessile papillae. The present SEM observation clearly showed the details of the cephalic structures, deirids, excretory pore, caudal papillae, vulva, phasmids and egg for the first time to our knowledge, which are helpful for the specific diagnosis of this species. *Physaloptera sibirica* is the fourth nematode parasite reported from *A. collaris*, which also represents a new host record for *P. sibirica*.

**Table 3:** Morphometric comparisons of *Physaloptera sibirica* (measurements in mm)

Characteristics	Present study		Petrow and Gorbunow [21]		Skrjabin and Sobolev [2]		Quentin and Biocca [24]		Li and Zhu [25]	
	Male	Female	Male	Female	Male	Female	Male	Female	Male	Female
BL	19.6–32.7	25.0–51.5	14.2‒16.3	18.7‒27.3	14.2‒16.3	18.7‒27.3	20	28.4	11.2–17.4	16.4–31.8
OL	3.46–5.37	4.39–7.32	–	–	–	–	4.80	5.65	–	–
ML	0.49–0.71	0.49–0.73	0.51‒0.76	0.62‒0.74	0.51‒0.76	0.62‒0.74	0.60	0.65	0.40–0.63	0.60–0.79
GL	3.00–4.88	3.90–6.59	3.06‒4.41	3.85‒5.55	3.06‒3.42	3.85‒5.55	4.20	5.00	3.00–3.40	3.50–5.50
ML/GL	1: 6.15–9.52	1: 8.00–10.5	–	–	–	–	1: 7.00	1: 7.69	–	–
OL/BL	14.4–18.9%	12.2–17.6%	–	–	–	–	24%	19.9%	–	–
TL	0.88–1.93	0.37–0.73	1.26‒1.45	0.49‒0.55	1.26‒1.45	0.49‒0.55	1.30	0.60	1.06–1.42	–
SL	0.54–1.27	N/A	0.63‒0.74	N/A	0.59‒0.74	N/A	0.78	N/A	0.64–0.80	N/A
SR	0.44–1.22	N/A	0.53‒0.64	N/A	0.53‒0.64	N/A	0.48	N/A	0.48–0.57	N/A
SL/BL	2.38–3.97%	N/A	–	N/A	–	N/A	3.9%	N/A	–	N/A
SR/BL	1.94–3.82%	N/A	–	N/A	–	N/A	2.4%	N/A	–	N/A
SR/SL	1: 1.04–1.25	N/A	1: 1.2	N/A	1: 1.2	N/A	1: 1.63	N/A	–	N/A
PP	4 pairs	N/A	4–5 pairs	N/A	5 pairs	N/A	4 pairs	N/A	4 pairs	N/A
PSP	3 precloacal, 5 pairs postcloacal	N/A	3 precloacal, 6 pairs postcloacal	N/A	3 precloacal, 6 pairs postcloacal	N/A	3 precloacal, 5 pairs postcloacal	N/A	3 precloacal, 5 pairs postcloacal	N/A
VC	N/A	4.10–9.81	N/A	2.95‒4.45	N/A	2.95‒4.45	N/A	4.06	N/A	3.54–4.10
VC/BL	N/A	13.9–21.4%	N/A	–	N/A	14.3%	N/A	14.3%	N/A	–
ES	N/A	0.04–0.05 × 0.02–0.04	N/A	0.047‒0.053 × 0.031–0.034	N/A	0.047‒0.053 × 0.031–0.034	N/A	0.047–0.05 × 0.032–0.034	N/A	0.05 × 0.035
Host	*Arctonyx collaris* (Mammalia: Carnivora)		*Nyctereutes amurensis*; *Vulpes vulpes* (Mammalia: Carnivora)		*Nyctereutes amurensis*; *Vulpes vulpes; Lynx lynx* (Mammalia: Carnivora)		*Eliomys quercinus* (Mammalia: Rodentia)		*Nyctereutes procyonoides* (Mammalia: Carnivora)	
Country	China (Shannxi Province)		Siberia; Far East; Novosibirsk		Far East; Siberia; Uzbekistan; Tatar		France, Italy		China (Heilongjiang Province)	

*BL* body length, *OL* length of oesophagus, *ML* length of muscular oesophagus, *GL* length of glandular oesophagus, *TL* length of tail, *SL* length of left spicule, *SR* length of right spicule, *PP* number of pedunculate papillae, *PSP* number and arrangement of caudal sessile papillae, *VC* distance of vulva to cephalic end, *ES* size of eggs

In the genus *Physaloptera*, there are 22 species with two uterine branches in females parasitic in mammals in the Palaearctic region, including *P. apodemi* Wang & Zhang, 2020; *P. clausa* Rudolphi, 1819; *P. anomala* Molin, 1860; *P. bedfordi* Ortlepp, 1932; *P. brevispiculum* Linstow, 1906; *P. brevivaginata* Seurat, 1917; *P. canis* Monnig, 1929; *P. dispar* Linstow, 1904; *P. getula* Seurat, 1917; *P. hispida* Schell, 1950; *P. lumsdeni* Yeh, 1957; *P. limbata* Leidy, 1856; *P. maxillaris* Molin, 1860; *P. murisbrasiliensis* Diesing, 1861; *P. massino* Schulz, 1926; *P. praeputiale* Linstow, 1889; *P. peramelis* Johnston & Mawson, 1939; *P. rara* Hall & Wigdor, 1918; *P. semilanceolata* Molin, 1860; *P. seurati* Isaitschikov, 1926, *P. sibirica* and *P. terdentata* Molin, 1860 [1, 2, 31‒35]. *Physaloptera sibirica* can be easily distinguished from *P. apodemi*, *P. anomala, P. brevispiculum, P. brevivaginata*, *P. dispar, P. getula, P. limbata, P. lumsdeni, P. maxillaris, P. massino*, *P. murisbrasiliensis, P. peramelis*, *P. praeputiale*, *P. rara* and *P. terdentata* by having unequal spicules (0.40–1.50 mm and right spicule < 1.7 times the left one in length) without striated sheaths at their proximal end and only four pairs of pedunculated caudal papillae. *Physaloptera sibirica* differs from *P. clausa*, *P. canis* and *P. bedfordi* by the position of the vulva (vulva from cephalic extremity representing about 1/7–1/5 of body length in *P. sibirica* vs. about 1/3–1/2 of the body length in the last three species). *Physaloptera sibirica* is different from *P. hispida* and *P. seurati* by having relatively longer spicules (spicules representing about 2.0‒4.0% of body length in *P. sibirica* vs. about 1.0% of body length in the latter two species). With two pairs of precloacal and two pairs of postcloacal pedunculated papillae, *P. sibirica* differs from *P. semilanceolata* (3 pairs precloacal and 1 pair postcloacal pedunculated papillae).

### Molecular characterization

#### Partial 18S region

Three 18S sequences of *P. sibirica* obtained herein are all 1710 bp in length, with no nucleotide polymorphism detected. In the genus *Physaloptera*, the 18S sequence data are available in GenBank for *P. alata* (AY702703), *P. amazonica* (MK312472), *P. apivori* (EU004817), *P. bispiculata* (KT894817), *P. mirandai* (KT894815, KT894816), *P. praeputialis* (MW410927), *P. rara* (MH938367), *P. retusa* (KT894814), *P. thalacomys* (JF934734) and *P. tupinambae* (MT810006). Pairwise comparison of the 18S sequences of *P. sibirica* with those of *Physaloptera* spp. available in GenBank displayed 0.11% (*P. rara*) to 2.10% (*P. alata*) of nucleotide divergence.

#### Partial ITS region

Three ITS sequences of *P. sibirica* obtained herein are 1272–1274 bp in length and represent two different genotypes, which showed 0.16% nucleotide divergence. In the genus *Physaloptera*, the ITS sequence data are available in GenBank only for *P. alata* (only 5.8S + ITS2 region, AY702694) and *P. tupinambae* (MT809124). Pairwise comparison of the ITS sequences of *P. sibirica* with those of *Physaloptera* spp. available in GenBank displayed 47.6% (*P. alata*) to 48.7% (*P. tupinambae*) nucleotide divergence.

#### Partial 28S region

Three 28S sequences of *P. sibirica* obtained herein are all 783 bp in length, with no nucleotide polymorphism detected. In the genus *Physaloptera*, the 28S sequence data are available in GenBank only for *Physaloptera* sp. (MG808041). Pairwise comparison of the 28S sequences of *P. sibirica* with that of *Physaloptera* sp. available in GenBank displayed 13.1% nucleotide divergence.

#### Partial cox1 region

Three *cox*1 sequences of *P. sibirica* obtained herein are all 892 bp in length, with no nucleotide polymorphism detected. In the genus *Physaloptera*, the *cox*1 sequence data are available in GenBank for *P. alata* (MZ391893), *P. amazonica* (MK309356), *P. bispiculata* (KT894806), *P. hispida* (MH782844, MH782845), *P. mirandai* (KP981418, KT894804, KT894805), *P. rara* (MH931178) and *P. retusa* (KT894803). Pairwise comparison of the *cox*1 sequences of *P. sibirica* with those of *Physaloptera* spp. available in GenBank displayed 15.2% (*P. rara*) to 19.5% (*P. bispiculata*) nucleotide divergence.

#### Partial cox2 region

Three *cox*2 sequences of *P. sibirica* obtained herein are all 376 bp in length and represent two different genotypes, which showed 2.39% nucleotide divergence. In the genus *Physaloptera*, the *cox*2 sequence data are available in GenBank only for *P. rara* (MH931178). Pairwise comparison of the *cox*2 sequences of *P. sibirica* with that of *P. rara* available in GenBank displayed 15.7% to 16.8% nucleotide divergence.

#### Partial 12S region

Three 12S sequences of *P. sibirica* obtained herein are all 472 bp in length, with no nucleotide polymorphism detected. In the genus *Physaloptera*, the 12S sequence data are available in GenBank only for *P. rara* (MH931178). Pairwise comparison of the 12S sequences of *P. sibirica* with that of *P. rara* available in GenBank displayed 14.8% nucleotide divergence.

### Phylogenetic analyses (Figs. 4, 5)

**Fig. 4:**
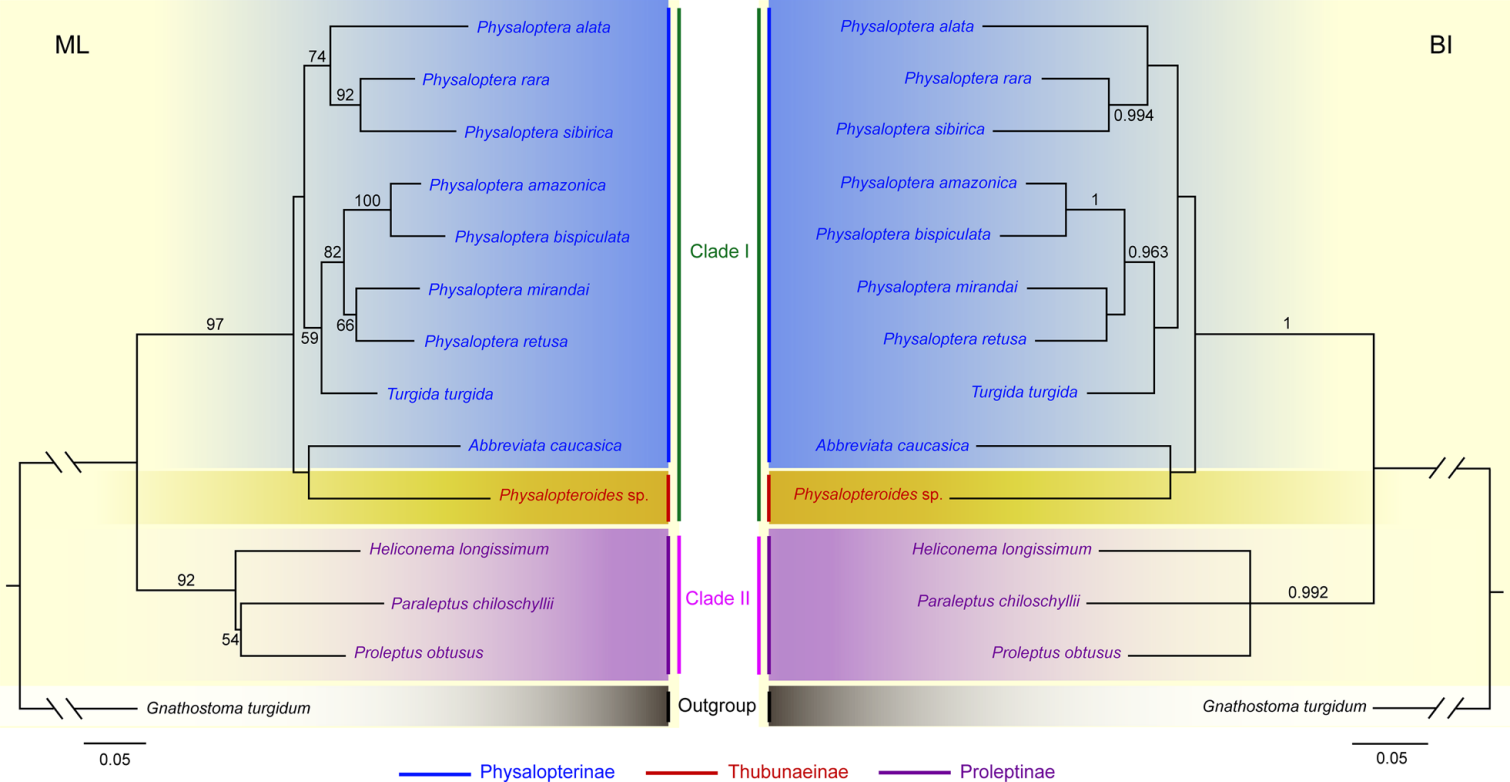
Maximum likelihood (ML) inference and Bayesian inference (BI) based on the 18S + *cox*1 sequence data showing the phylogenetic relationships of representatives of Physalopteridae. *Gnathostoma turgidum* Stossich, 1902 (Spirurida: Gnathostomatidae), was chosen as the outgroup. Bootstrap support (BS) values ≥ 50 in ML tree and Bayesian posterior probabilities (BPP) ≥ 0.95 in BI tree are shown

**Fig. 5:**
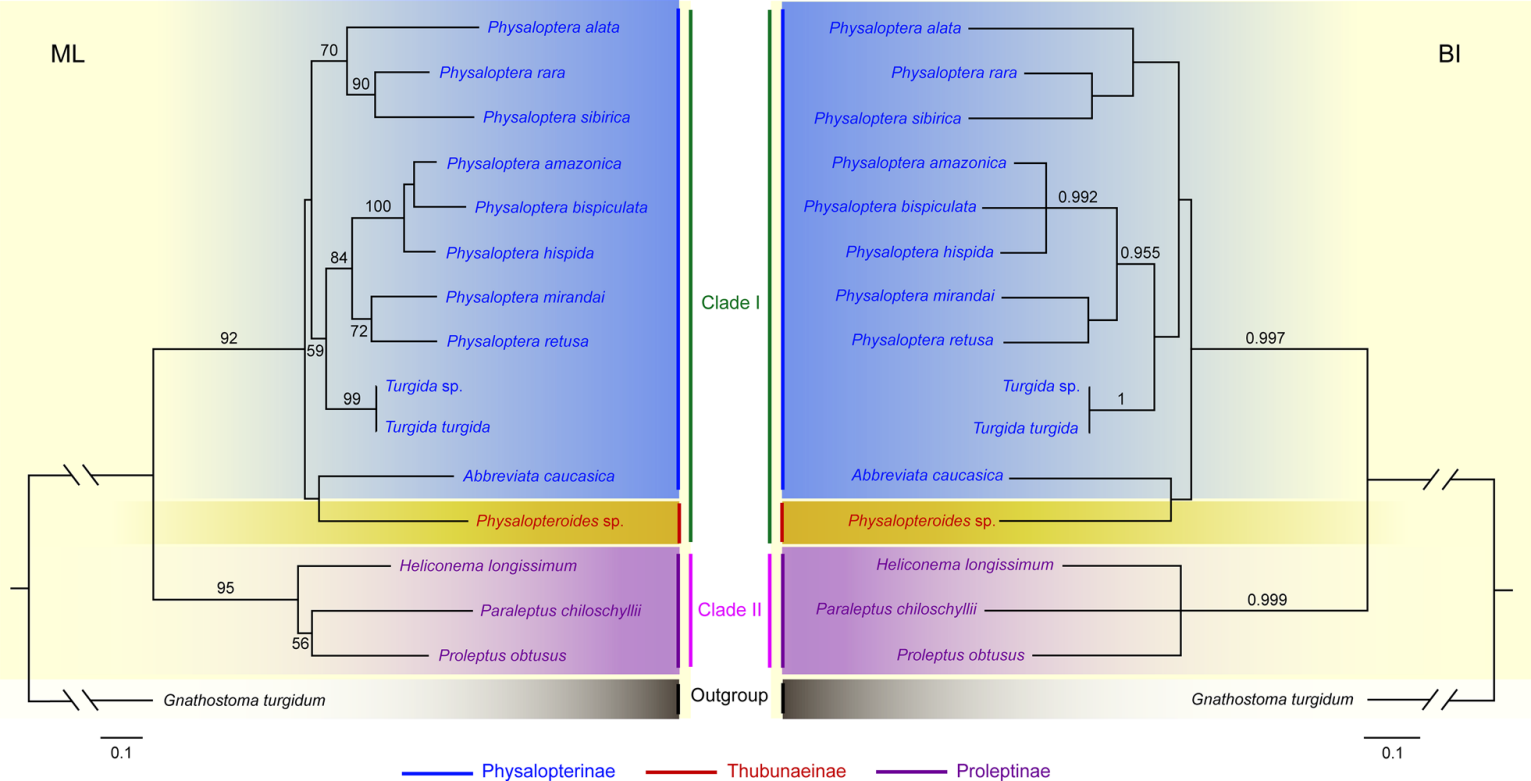
Maximum likelihood (ML) inference and Bayesian inference (BI) based on the *cox*1 sequence data showing the phylogenetic relationships of representatives of Physalopteridae. *Gnathostoma turgidum* Stossich, 1902 (Spirurida: Gnathostomatidae), was chosen as the outgroup. Bootstrap support (BS) values ≥ 50 in ML tree and Bayesian posterior probabilities (BPP) ≥ 0.95 in BI tree are shown

Phylogenetic trees constructed from the 18S + *cox*1 and *cox*1 sequence data using the ML and BI methods had almost identical topologies (Figs. 4–5). The representatives of the Physalopteridae were divided into two major clades. Clade I included species of *Physaloptera*, *Turgida* and *Abbreviara* (Physalopterinae), and *Physalopteroides* (Thubunaeinae). Clade II contained representatives of *Heliconema*, *Paraleptus* and *Proleptus* (Proleptinae). In Clade I, *P. alata* + (*P. rara* + *P. sibirica*) formed a separated branch with moderate support in ML tree, but weak support in BI tree (Figs. 4, 5). *Turgida turgida* clustered together with *P. amazonica* + *P. bispiculata* + *P. mirandai* + *P. retusa*. *Physalopteroides* sp. (Thubunaeinae) formed a sister relationship to *Abbreviata caucasica* (Physalopterinae).

## Discussion

Skrjabin (1964, 1969) [2, 31] assigned the genus *Thubunaea* into the subfamily Proleptinae as a tribe Thubunaeinea. Later, Chabaud (1975) [6] treated Thubunaeinea as a separated subfamily Thubunaeinae, including only two genera, *Thubunaea* and *Physalopteroides*. Chabaud & Bain (1994) [7] speculated that in the family Physalopteridae, the Physalopterinae and Proleptinae had closer relationships than the Thubunaeinae based on the cephalic structures. However, our phylogenetic results challenged the validity of the subfamily Thubunaeinae because of the single representative of Thubunaeinae (*Physalopteroides* sp.) clustered together with species of *Abbreviata* (member of Physalopterinae) and supported the classification of Physalopteridae comprising only two subfamilies, Physalopterinae and Proleptinae, proposed by Skrjabin & Sobolev (1964) [2], which conflicted with the above-mentioned traditional opinions [6, 7]. We considered that the features of cephalic collarette and caudal bursa used as the main criterion for differentiating the Thubunaeinae from Physalopterinae and Proleptinae by Chabaud (1975) [6] are rather vulnerable and questionable. The division of Physalopteridae into Physalopterinae and Proleptinae can be easily understood when we consider the host range and geographical distribution of the species in Physalopterinae, Proleptinae and Thubunaeinae (species of Physalopterinae and Thubunaeinae are both parasitic in terrestrial vertebrates vs. species of Proleptinae occur only in teleosts and elasmobranchs). Wason & Johnson (1977) [36] erected a subfamily Mirzalopterinae for the genus *Mirzaloptera* (type species *M. barbari* Wason & Johnson, 1977, collected from bat in India) in the Physalopteridae. However, the Mirzalopterinae has received little attention since its inception, and only the compilations of Jones & Gibson (1987) [37] and Gibbons (2010) [38] included it. The systematic status of Mirzalopterinae has been unclear.

The present molecular analyses also challenged the validity of the genus *Turgida*, because *T. turgida* nested among representatives of *Physaloptera*, which are accordant with previous phylogenetic studies [18, 19, 39]. In fact, Ortlepp (1922) [1] and Yorke & Maplestone (1927) [40] both suspected the validity of *Turgida*. Although Travassos (1920) [41] used the uterus with 10 uterine branches as a generic criterion in separating *Turgida* from the other genera in the Physalopterinae, the number of uterine branches in the type species of *Turgida*, *T. turgida* seems to be extremely variable. Gray & Anderson (1982) [42] observed 7 to 10 uterine branches in their specimens collected from opossum *Didelphis virginiana*. Ortlepp (1922) [1] reported that there were up to 14 uterine branches in female of *T. turgida*. Meanwhile, species of *Physaloptera* have also been reported to exhibit a broad range of variability in a number of uterine branches among different species, for example, *P. ackerti* and *P. aduensis* possessing six to nine uterine branches; *P. amazonica* and *P. goytaca* having four or five uterine branches; *P. clausa* and *P. apodemi* having two uterine branches [1, 4, 18, 32, 33, 43‒45]. The variability in the number of uterine branches among different individuals of *T. turgida* and different species of *Physaloptera* brings into question the reliability of the number of uterine branches as a generic criterion. We considered that the number of uterine branches should be treated as a specific character. The present phylogenetic results indicated that the number of uterine branches of *Physaloptera* species is not in relation to the relationships of species in the phylogenetic trees, because *T. turgida* with 7‒14 uterine branches and *P. amazonica* with 4 uterine branches are scattered into the other *Physaloptera* species with ony two uterine branches (including *P. bispiculata*, *P. mirandai*, *P. retusa*, *P. alata*, *P. rara* and *sibirica*). In contrast, our results showed that the phylogenetic relationships of *Physaloptera* species seem to be associated with their geographical distribution, because all species from the Neotropical region (Brazil) (including *P. amazonica*, *P. bispiculata*, *P. mirandai*, *P. retusa* and *T. turgida*) formed a monophyletic subclade, which displayed a sister to the other subclade constituted by the species from the Palearctic region (*P. alata* and *P. sibirica*) and Palearctic + Nearctic region (*P. rara*).

The present molecular phylogenies reinforced the limited knowledge pertaining to the evolution of physalopterid nematodes. However, we do not make any immediate systematic changes in the Physalopteridae, and care must be taken in using the preliminary phylogenetic results, because only a limited number of physalopterid representatives were included in the phylogeny, and many of their genetic data have not been critically evaluated. Overall, a more rigorous molecular phylogeny including species of *Thubunaea* and broader representatives of *Turgida, Physaloptera* and *Physalopteroides* is required to further test the systematic status of Thubunaeinae and *Turgida* and clarify the phylogeographic pattern of *Physaloptera*.

Pairwise comparison of the sequences obtained for *P. sibirica* revealed no genetic divergence regarding the 18S, 28S, *cox*1 and 12S genetic markers and a low level of divergence in the ITS (0.16%) and *cox*2 (2.39%) regions. The high genetic divergence observed between the present specimens and other species of *Physaloptera* further confirmed their distinct specific identity. The genetic data presented here will contribute to the molecular identification, population genetics and phylogenetics of physalopterid nematodes.

## Conclusions

The detailed morphology of *P. sibirica* was further studied using light and scanning electron microscopy based on newly collected specimens from the hog badger *A. collaris* in China. *Physaloptera sibirica* is the fourth nematode parasite reported from the hog badger* A. collaris*, and *A. collaris* represents a new host for *P. sibirica*. The characterization of the nuclear 18S, 28S and ITS and mitochondrial *cox*1, *cox*2 and 12S sequences of *P. sibirica* were provided for the first time. The phylogenetic results indicated that there could be issues with the current understanding of the systematic status of the subfamily Thubunaeinae and the genus *Turgida* within the Physalopteridae, and more genetic data are required across the species and genera that do not yet have molecular information to further clarify the phylogenetic relationships of the three subfamilies Thubunaeinae, Physalopterinae and Proleptinae. These present findings contribute to morphologically recognizing *P. sibirica* more accurately and provide new insights into the systematics of the family Physalopteridae.

## Data Availability

The nuclear and mitochondrial DNA sequences of *Physaloptera sibirica* obtained in the present study were deposited in GenBank database under the accession numbers OQ846900–OQ846902 (18S), OQ846911–OQ846913 (ITS), OQ846907–OQ846909 (28S), OQ852731–OQ852733 (*cox*1), OQ867994–OQ867996 (*cox*2) and OQ846904–OQ846906 (12S). Specimens of *Physaloptera sibirica* were deposited in the College of Life Sciences, Hebei Normal University, Hebei Province, China, under accession number HBNU–N-2022M1120-CL.

## Abbreviations

SEM: Scanning electron microscopy
PCR: Polymerase chain reaction
ML: Maximum likelihood
BI: Bayesian inference
BIC: Bayesian information criterion
18S: Small ribosomal subunit
28S: Large ribosomal subunit
ITS: Internal transcribed spacer
*cox*1: Cytochrome *c* oxidase subunit 1
*cox*2: Cytochrome *c* oxidase subunit 2
12S: 12S small subunit ribosomal RNA gene
